# A Pyogenic Liver Abscess in the Setting of Fontan-Associated Liver Disease

**DOI:** 10.7759/cureus.98255

**Published:** 2025-12-01

**Authors:** Thomas J Mason, John A Gambril, Joshua Spegman, Matthew Laubham

**Affiliations:** 1 Internal Medicine - Pediatrics, The Ohio State University, Nationwide Children's Hospital, Columbus, USA; 2 Cardiology, University of Alabama at Birmingham School of Medicine, Birmingham, USA; 3 Adult Congenital Heart Disease, The Ohio State University, Nationwide Children's Hospital, Columbus, USA

**Keywords:** adult congenital heart disease (achd), congenital heart diseases (chds), congestive hepatopathy, fontan-associated liver disease, fontan circulation, fontan physiology, pyogenic liver abscess (pla)

## Abstract

The Fontan procedure is widely used today in the surgical management of congenital heart disease as the final step in single ventricle palliation pathways. It is associated with unique complications, most notably Fontan-associated liver disease (FALD), which is a condition arising from the unique hemodynamics of the Fontan circulation that occurs universally after the procedure. Though varying in severity from mild hepatocellular liver injury to frank decompensated cirrhosis, FALD progresses at different rates depending on the patient. Given the significant morbidity and mortality associated with late-stage FALD, a better understanding of the pathophysiology and complications is needed. Here we describe a unique case of a pyogenic liver abscess in a patient with FALD, possibly the first reported in the literature.

## Introduction

In 1971, Francis Fontan pioneered the first total subpulmonic ventricular bypass procedure to improve cyanosis in a patient with tricuspid atresia. After various modifications, the eponymous Fontan procedure has become the standard of care in the management of single ventricle congenital heart disease as the final step in surgical palliation [1]. Briefly, the Fontan procedure creates a pathway for lower extremity venous blood to bypass the heart and flow directly into the pulmonary arteries. When coupled with a Glenn shunt, which allows the flow of upper extremity venous blood into the pulmonary arteries, the Fontan procedure allows complete passive cavopulmonary blood flow. This serves to improve cyanosis and systemic ventricular volume load in patients with congenital single ventricle heart disease of many forms. It is estimated that 50,000-70,000 people worldwide are living with Fontan circulation, with 40-55% of these being adults [2,3]. Surgical and transcatheter advancements, as well as improved medical management of Fontan-associated comorbidities, have greatly improved survival, with a contemporary estimated 30-year survival rate of 85% [2].

With improved survival, the frequency of newer complications has increased, like Fontan-associated liver disease (FALD). FALD encompasses the spectrum of functional and structural alterations of the liver, including synthetic dysfunction and fibrosis, and if progressive, frank cirrhosis, portal hypertension, and hepatocellular carcinoma (HCC) [4]. The pathophysiology of FALD includes elevated systemic venous pressure leading to inefficient blood drainage from the liver, creating chronic passive congestion and noninflammatory fibrogenesis. Clinical presentation of FALD is variable, but can be characterized by elevated liver enzymes, abnormal hepatocyte synthetic function, esophageal or gastric varices, splenomegaly, ascites, and encephalopathy, depending on the degree of liver disease and the presence of portal hypertension [5,6]. Unfortunately, hepatic fibrosis develops universally after Fontan palliation, with current estimates that 30% of patients will develop cirrhosis by 15 years postoperatively [7]. Though differing in etiology, the end-stage complications of FALD are similar to those of decompensated cirrhosis from other causes [8]. However, given the limited number of patients with FALD, rare complications are now arising that are thought to be related to the unique pathophysiology of the Fontan circulation. A better understanding of these cases will allow the development of strategies to avoid additional hepatic insults and improve long-term morbidity and mortality in patients with invariably progressive FALD. After an extensive literature search, here we detail what is possibly the first reported case of a pyogenic liver abscess in the setting of FALD.

## Case presentation

We describe a 40-year-old male who presented with one week of fevers and malaise and, acutely, one day of emesis and diarrhea. His past medical history was notable for a double inlet left ventricle with malposed great arteries and subvalvular pulmonary stenosis that was ultimately palliated with a lateral tunnel nonfenestrated Fontan. Known complications since include moderate systemic ventricular dysfunction as well as FALD with some imaging evidence of cirrhosis. After presenting with worsening malaise and fevers, he was found to be hypotensive and in shock with a lactate of 9 mmol/L and evidence of multiorgan dysfunction. His inflammatory markers were notably elevated with a significant leukocytosis, and his blood culture grew *Streptococcus intermedius* (Table 1). He was aggressively fluid resuscitated, started on vasopressors for persistent shock, and treated with broad-spectrum antibiotics. He demonstrated slow but steady hemodynamic improvement over several days.

**Figure 1:**
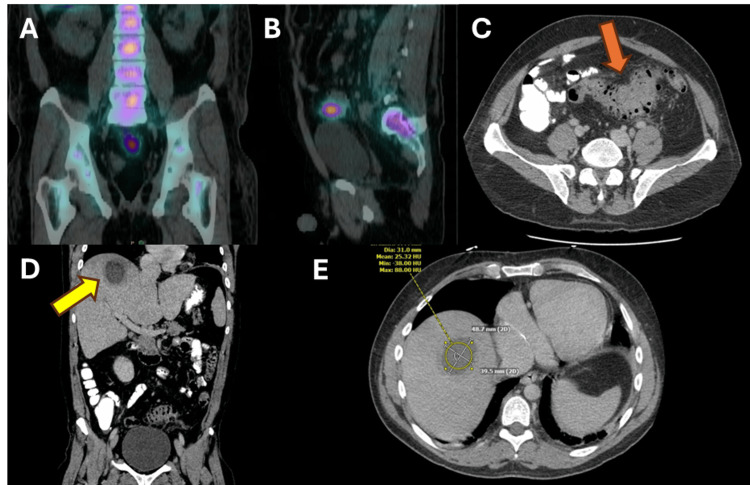
Imaging Studies of the Hepatic Abscess and Diverticulitis Indium-111 radionuclide scan demonstrating the area of rectosigmoid inflammation seen initially in coronal (A) and sagittal (B) views. When the patient re-presented, his computed tomography (CT) scan of the abdomen and pelvis demonstrated diverticulitis without frank abscess (red arrow) in the area previously highlighted on the radionuclide scan (C) and a 4 cm by 5 cm hepatic dome abscess (yellow arrow) seen in coronal (D) and transverse (E) views.

A broad workup for a source of his bacteremia was undertaken, including urine cultures, an echocardiogram, and dental X-rays, but all were unrevealing. Given the high clinical concern that he may have an infected piece of intracardiac prosthetic material, an Indium-111 tagged white blood cell scan was pursued, though this did not show any uptake in the heart and only demonstrated abnormal uptake in the sigmoid colon and rectum with a radiographic appearance consistent with colitis or diverticulitis (Figure 1).

After demonstrating clinical stability and having complete resolution of his fevers, malaise, emesis, and diarrhea, he was discharged on a six-week course of ceftriaxone for presumed endocarditis, given the lack of a clear source for his bacteremia. He re-presented, however, four days later with new fevers and recurrent septic shock. Laboratory values were notable for recurrent leukocytosis with elevated but lower inflammatory markers than his previous presentation (Table 1). CT imaging demonstrated severe sigmoid diverticulitis and interval development of a hepatic dome abscess (Figure 1). He briefly required vasopressors but responded rapidly to broad-spectrum antibiotics and percutaneous drainage of his hepatic dome abscess. Cultures from blood and abscess were negative, so he was ultimately discharged to complete a six-week course of intravenous ceftriaxone 2 grams daily, now with metronidazole 500 mg three times daily. His rapid improvement after drainage of his liver dome abscess suggested that the interval development of this abscess caused his recurrent presentation as opposed to a resistant organism. He ultimately had persistent sigmoid diverticulitis after discharge that was complicated by abscess formation and underwent an uncomplicated laparoscopic sigmoidectomy about eight months after his initial presentation. Since then, he has been doing well with no further infectious issues.

**Table 1:** Objective Data by Hospitalization Notable vital signs and laboratory values on initial presentation for our patient during his back to back hospitalizations. His baseline creatinine was < 1.0 and his baseline oxygen saturation (SpO_2_) was >90% when breathing ambient air. All oxygen saturations noted are while breathing ambient air. HR: heart rate, bpm: beats per minute, BP: blood pressure, RR: respiratory rate, rpm: respirations per minute, NT: not tested, NG: no growth.

Test	Reference	Hospitalization #1	Hospitalization #2
Vital Signs		Temp 98.2 °F, HR 101 bpm, BP 80/40 mm Hg, RR 16 rpm, SpO_2_ 96%	Temp 99.2 °F, HR 108 bpm, BP 72/42 mm Hg, RR 18 rpm, SpO_2_ 92%
Lactate (mmol/L)	0.5-1.6	9	1.6
Creatinine (mg/dL)	0.7-1.3	2.6	1.3
White blood cell count (k/µL)	3.7-10.1	18	21
C-reactive protein (mg/L)	<10	296	NT
Procalcitonin (ng/mL)	<0.5	235	2.1
Blood culture	NG	Streptococcus intermedius	NG

## Discussion

This case demonstrates a rare but highly consequential complication of FALD. The development of a pyogenic liver abscess secondary to an intra-abdominal infection in our patient raises an important point regarding the relationship between Fontan circulation and the intra-abdominal abscess. FALD occurs due to a combination of acute and chronic liver injuries related to the elevated central venous pressure (CVP) required to achieve passive cavopulmonary blood flow in the Fontan circulation. This elevated CVP is directly transmitted into the hepatic veins, resulting in chronic congestive hepatopathy and hepatic sinusoidal dilation. The insult can be furthered by baseline hypoxemia and depressed systemic ventricular function that is variably seen in patients depending on their native physiology and fenestration status, resulting in superimposed chronic ischemic hepatitis. Additionally, there are multiple acute hepatic insults that occur during the perioperative period and due to poor circulatory compensation during periods of acute illness, as seen in our patient. This multifactorial process invariably results in progressive hepatic fibrosis, the severity of which is directly correlated with time from Fontan completion [5,6].

The source of this patient’s bacteremia was almost certainly the sigmoid diverticulitis that became clinically apparent during his second admission and beyond. Bacteremia with *Streptococcus intermedius* as the culprit organism supports this theory, given that most species of viridans streptococci are typical gastrointestinal flora with the potential to translocate into the bloodstream [9]. This favors the hypothesis that the development of the liver abscess after the first hospitalization caused the recurrent presentation of shock. In the general population, there is evidence that cirrhosis increases the risk of pyogenic liver abscess development, though this is difficult to attribute exclusively to portal hypertension in the setting of the immunocompromised state conferred by cirrhosis [10,11]. While our patient had CT evidence of possible cirrhosis, notably with reticular heterogeneity of the hepatic parenchyma and a nodular liver edge, these findings are nonspecific in the setting of FALD, and the degree of fibrosis is notoriously difficult to stage based on imaging alone [6,12-13]. Thus, it is difficult to definitively say that he had a pathological diagnosis of cirrhosis, especially given that he did not have any radiographic or clinical evidence of portal hypertension and had uncompromised hepatic synthetic function.

We feel it is more likely that the chronic hepatic congestion conferred by his Fontan circulation and mild systemic ventricular dysfunction placed him at increased risk for bacterial translocation through his chronically dilated hepatic sinusoids. In this hypothesis, bacteria translocated from the focal rectosigmoid infection through the portal vasculature and seeded the hepatic parenchyma due to sluggish blood flow and endothelial dysfunction in the hepatic sinusoids. It is possible that this occurred during his first episode of shock, given the additional acute insult this applied to his hepatic sinusoidal endothelium and further slowing of hepatic blood flow. Based on limited data, a small abscess in the liver could have been missed on the Indium-111 tagged white blood cell scan due to reduced sensitivity compared to CT [14]. Based on a thorough review of the literature, we believe this is the only case of FALD and an associated hepatic abscess reported.

## Conclusions

Given the increasing prevalence of adult patients with Fontan palliations and their significantly improved survival over the past 30 years, a better understanding of the pathophysiology of FALD is needed to elucidate prevention strategies and improve current surveillance strategies for the disease process. This is particularly important in patients with Fontan circulation, as most will ultimately require a heart transplant when their single ventricle fails. Significant liver disease in this setting generates additional morbidity that can preclude heart transplantation outright or creates a need for an even more technically challenging heart-liver transplant. Thus, prevention strategies for avoiding hepatic insults and slowing the progression of FALD are necessary to allow these patients to live longer and healthier lives.

As with some of the more common complications of liver disease, understanding the unique complications of the disease process may allow us to better appreciate the pathophysiology and potential prevention strategies. Our case describes a rare presentation of a pyogenic liver abscess thought to be caused by the elevated portal pressures and sinusoidal dilation seen in FALD. Understanding the underlying mechanisms of sinusoidal dilation in the face of elevated portal pressures could allow the development of hepatoprotective drug therapy for FALD and more robust perioperative management strategies for avoiding acute hepatic insults that contribute to the development of the disease. All said, further work is needed to understand the pathogenesis and unique complications of FALD to better prevent and manage the disease.
